# The effect of anaesthesia technique on caesarean section

**DOI:** 10.12669/pjms.321.9028

**Published:** 2016

**Authors:** Young Duck Shin, Sang Hi Park, Hyeon Tae Kim, Chan Jin Park, Jin Hee Lee, Young Jin Choi

**Affiliations:** 1 Young Duck Shin, Department of Anesthesiology and Pain Medicine, College of Medicine, Chungbuk National University, Korea; 2 Sang Hi Park, Department of Anesthesiology and Pain Medicine Chungbuk National University Hospital, Korea; 3 Hyeon Tae Kim, Department of Anesthesiology and Pain Medicine Chungbuk National University Hospital, Korea; 4 Chan Jin Park, Department of Anesthesiology and Pain Medicine Chungbuk National University Hospital, Korea; 5 Jin Hee Lee, Department of Anesthesiology and Pain Medicine Chungbuk National University Hospital, Korea; 6 Young Jin Choi, Department of Surgery, College of Medicine, Chungbuk National University, Korea

**Keywords:** Anaesthesia, Caesarean section, Epidural, Spinal

## Abstract

**Objective:**

When conducting a caesarean section under regional anaesthesia, either epidural anaesthesia or spinal anaesthesia can be used. Patients who underwent caesarean section in our hospital were surveyed retrospectively to confirm and compare the merits and demerits of spinal anaesthesia and epidural anaesthesia to determine the most efficient approach.

**Methods:**

Mothers meeting the American Society of Anesthesiologists physical status classification system (ASA) I or II, who underwent caesarean sections at our hospital were surveyed retrospectively. The survey targeted one hundred patients each who received spinal anaesthesia and epidural anaesthesia. The time from anaesthesia to surgical incision (A to S time), entire anaesthesia time, and the usage of vasopressor and midazolam were compared according to anaesthetic approach.

**Results:**

The A to S time and the entire anaesthesia time of the group that underwent spinal anaesthesia were significantly short compared to the times recorded for the group who underwent epidural anaesthesia, and the use of vasopressor was more frequent in the spinal anaesthesia group because their blood pressure decline was larger.

**Conclusion:**

The A to S time and the entire anaesthetic time were longer for epidural anaesthesia than for spinal anaesthesia. However, the haemodynamic change was smaller and vasopressor was hardly used in the former group. Therefore, the choice of the technical method will depend on the clinical, anaesthetic, and obstetric situation.

## INTRODUCTION

A 2001 obstetric anaesthesia workforce survey in United States revealed that most patients of caesarean section have this under spinal or epidural anaesthesia.^1^ Regional anaesthesia, compared to general anaesthesia, reduces the risk of pulmonary aspiration and airway problems arising due to intubation failure.^2^ Epidural anaesthesia is known to be able to induce anaesthesia without causing a sudden cardiovascular change in the case of haemodynamic instability, while spinal anaesthesia is easier and faster than epidural anaesthesia and allows a reduction of anaesthesia-induction time.^3^ However, there is a need to assess the relative efficacy and side-effects of regional anaesthesia in women undergoing caesarean section, because anaesthesia for caesarean section is still far from ideal. The choice of anaesthesia depends on foetal and maternal conditions, pregnant women’s and anaesthesiologists’ preferences, and the indications of the surgery.^2^

In this study, the merits and demerits of spinal anaesthesia and epidural anaesthesia were confirmed and compared by a patient survey, in order to identify the most efficient method in terms of the reduction of operation time and haemodynamic stability.

## METHODS

The present study was approved by the Institutional Review Board (IRB) of Chungbuk National University Hospital (CBNUH 2015-10-015). The inclusion criteria were mothers meeting the American Society of Anesthesiologists physical status classification system (ASA) I or II, who underwent caesarean sections, including routine and emergency operations. One hundred patients each who underwent spinal anaesthesia or epidural anaesthesia were targeted; the relevant data were surveyed retrospectively, and hence no power calculation was performed. The exclusion criteria were patients of ASA III-V classification, or who underwent general anaesthesia.

The patients did not receive any premedication. For intraoperative monitoring, ECG, non-invasive blood pressure measurements, and pulse oximetry were included. Oxygen at 5 L/min was applied via a mask. Before anaesthesia, patients were administered 400−500 mL of lactated Ringer’s solution. Epidural anaesthesia was carried out by adding fentanyl (100 mcg) to 0.75% levobupivacaine (15−25 mL), through the loss-of-resistance-to-air technique. Anaesthetic was administered between lumbar vertebrae 3 and 4, using an 18-gauge Tuohy needle and a 20-gauge catheter, with the patient in the sitting position. Spinal anaesthesia was carried out using a 26-gauge spinal needle, and involved adding 0.5% bupivacaine (10−12 mg) and fentanyl (10−20 mcg) to the same interspace. The age, gestational age, height, weight, and ASA status of the targeted patients were surveyed, and it was monitored whether systolic blood pressure declined more than 20% as compared to the baseline. The skin sensory block degree was recorded after administering the regional anaesthesia, and the time from anaesthesia to surgical incision (A to S) time, entire anaesthesia time, usage of ephedrine or phenylephrine, and midazolam usage were compared according to the anaesthesia approach used. The one minute and five minute. Apgar score of the newborn, the visual analogue scale (VAS) pain score at one day after surgery, and the state of the postdural puncture headache (PDPH) were also compared.

### Statistical analysis

The age, weight, height, anaesthesia time of patients, usage of drugs, Apgar score, and VAS pain score are shown as mean ± standard deviation values, and SPSS version 12.0 was used for statistical analysis. The unpaired *t*-test was used to compare the two groups and the chi- square test was used for frequency examination. P-values < 0.05 were considered significant.

## RESULTS

There was no significant difference in the age, height, and weight of the patients in the two groups, and there was also no difference in sensory block level and ASA status (Table-I). However, there was a significant difference in the A-to-S time, the entire anaesthesia time, and the degree of use of ephedrine or phenylephrine between the two groups. Both A-to-S and total anaesthesia times were shorter in the spinal anaesthesia group than in the epidural anaesthesia group.

**Table I:** Demographic data of patients.

	Spinal anaesthesia	Epidural anaesthesia	p-value
Age (y)	34.1 ± 3.75	34.5 ± 4.10	0.69
Height (cm)	157.87 ± 5.87	157.55 ± 7.56	0.55
Body weight (kg)	73.60 ± 11.05	72.85 ± 10.31	0.78
ASA (I/II)	83/17	90/10	0.70
Block level	5.73	5.23	0.32

Data are presented as N or mean ± SD.

However, systolic blood pressure decreased more and the use of vasopressor was more frequent in the spinal than in the epidural anaesthesia group (Table-II). There was no significant difference between the groups with respect to the one minute of five -minute Apgar score of the newborn, the VAS pain score at 1 day after surgery, and the PDPH degree (Table-III).

**Table II:** Perioperative events for different anaesthesia approaches.

	Spinal anaesthesia	Epidural anaesthesia
A-to-S time (min)	20.41 ± 3.77	27.5 ± 5.67*
Total anaesthetic time (min)	84.63 ± 16.87	90.87 ± 15.58*
SBP decrease > 20%	40.8%	23.5%*
Ephedrine/phenylephrine use	65.8%	30.76%*
Ephedrine (mg)	8.4 ± 3.6	3.6 ± 2.7*

Data are presented as N or mean ± SD, unless otherwise indicated.

* P < 0.05 compared with spinal anaesthesia.

**Table III:** Newborn Apgar scores and maternal pain scores after caesarean section.

	Spinal anaesthesia	Epidural anaesthesia
Apgar score (at 1 min)	8.80 ± 0.87	9.10 ± 0.65
Apgar score (at 5 min)	9.20 ± 0.79	9.60 ± 0.29
VAS pain scores on postoperative day 1	2.75 ± 1.42	3.20 ± 1.56

Data are presented as N or mean ± SD, unless otherwise indicated. VAS: visual analogue scale. * P < 0.05 compared with spinal anaesthesia

## DISCUSSION

Anaesthesia during caesarean section can eliminate pain and shows few side effects in the mother and infant. Therefore, in obstetrics, the ideal is that anaesthesia time should be as short as possible and that the haemodynamic changes should be minimized in order to maintain the blood flow through the uterus. Since the maternal mortality rate under general anaesthesia is 16 times as high as that for regional anaesthesia,^4^ regional anaesthesia is preferred to general anaesthesia for patients undergoing caesarean section.^5^ Generally, spinal anaesthesia allows a fast induction of anaesthesia, and enhances the turnover rate in the theatre, compared to epidural anaesthesia.^6^ Since surgeons tend to believe that is preferable for the infant to be taken out as quickly as possible, several hospitals administer spinal anaesthesia, even though an epidural catheter is inserted with a view to painless vaginal delivery.^7^

The time from commencing anaesthesia to the start of surgery and the entire anaesthesia time was significantly shorter with spinal anaesthesia, which is advantageous. However, the systolic blood pressure more often decreased > 20% compared to baseline after this form of anaesthesia; therefore, the frequency of use and the amount of vasopressor, such as ephedrine or phenylephrine, used were also greater with spinal anaesthesia. Although the anaesthesia level of both anaesthetic techniques used in this study appeared similar, with an average of T5, in a previous study, the level of anaesthesia increased quickly with spinal anaesthesia, and respiratory insufficiency or unconsciousness occurred, such that even total spinal anaesthesia and conversion into general anaesthesia with intubation has been reported.^8^

The merits and demerits of spinal anaesthesia and epidural anaesthesia stand in clear contrast, yet, the use of combined spinal and epidural anaesthesia has become common recently. Since the combined spinal epidural anaesthesia shares the advantages of epidural anaesthesia of inducing spinal anaesthesia quickly and reinforcing intermediate blockage, complications, such as high-level blockage or hypotension, can be reduced by decreasing the volume of the spinal anaesthetic used.^9^ However, as with epidural anaesthesia, the anaesthesia time is longer than with spinal anaesthesia only, and the level of the anaesthesia increases as fast as in spinal anaesthesia, so that there is also the drawback of rapid haemodynamic changes. According to a recent study, the possibility of failure was higher for the combined anaesthesia than for spinal anaesthesia alone; the likelihood of converting into general anaesthesia after failure of epidural anaesthesia was 5%, re-attempt was 7.74%, and the requirement for other sedatives or analgesics during surgery was 10.74%.^10^

In this study, there was no difference between the groups in terms of the state of the newborn baby and the pain experienced by the patient after surgery. Considering these results, it is proposed that, while general anaesthesia should be used when the foetal state worsens rapidly, spinal anaesthesia should be used in cases of relative urgency. Epidural anaesthesia should be used with careful monitoring of haemodynamic changes in those cases where the patient’s and foetus’s state is stable.

## CONCLUSION

The time from anaesthesia to the start of surgery and the entire anaesthetic time were longer with epidural anaesthesia than with spinal anaesthesia. However, the haemodynamic changes were small and vasopressor use was minimal in both groups. Furthermore, the Apgar score of the newborn baby was similar in the two groups, thus the type of anaesthesia used had no different effects on the newborn. Accordingly, the choice of anaesthesia technique used will depend on the clinical, anaesthetic, and obstetrical situation in each case.
